# The influence of grain shape and size on the relationship between porosity and permeability in sandstone: a digital approach

**DOI:** 10.1038/s41598-022-11365-8

**Published:** 2022-05-09

**Authors:** Ryan L. Payton, Domenico Chiarella, Andrew Kingdon

**Affiliations:** 1 Department of Earth Sciences, Clastic Sedimentology Investigation (CSI), Royal Holloway, University of London, Egham, Surrey UK; 2 British Geological Survey, Keyworth, Nottingham UK

**Keywords:** Geology, Geophysics, Sedimentology

## Abstract

An accurate and reliable description of the porosity–permeability relationship in geological materials is valuable in understanding subsurface fluid movement. This is important for reservoir characterisation, energy exploitation, geological carbon storage (GCS) and groundwater contamination and remediation. Whilst the relationship between pore characteristics and porosity and permeability are well examined, further investigation into the influence of grain characteristics on porosity and permeability would be beneficial due to the inherent relationship between grains and pores. This work aims to determine whether incorporation of grain characteristics into a porosity–permeability model is effective in constraining this relationship. Two fully digital approaches to individual 3D grain analysis based upon watershed segmentation are compared to determine the most effective, yet simple, workflow applicable to core plugs of significantly compacted grains. The identification of an effective segmentation workflow will facilitate future work on similarly complex materials, removing the need for traditional time-consuming and manual techniques. We use the most effective approach of measuring grain shape (sphericity) and size (Feret diameter) alongside an established fully digital workflow to measure porosity and permeability to investigate the impact of grain characteristics on porosity and permeability. We show that grain sphericity and porosity exhibit a positive relationship whereas no such relationship exists with grain size. Measurements of grain sphericity are applied to calculate a Kozeny–Carman (K–C) type porosity–permeability fit which was found to be unsatisfactory, compared to a simpler fit excluding any grain parameters. This is possibly due to the lower sphericity of the studied grains, deviating significantly from the K–C assumption that grains are entirely spherical. The simpler fit is most suitable for the studied materials, showing that inclusion of grain characteristics is not effective for better defining the porosity–permeability relationship in a K–C paradigm for these samples. This highlights the need for a model capable of considering a range of grain sphericities to further constrain the porosity–permeability relationship.

## Introduction

The relationship between porosity and permeability is very significant for reservoir characterisation studies applied to geological carbon storage, energy resource exploitation, and aquifer contamination and remediation. Constraining the relationship between these two important reservoir parameters is beneficial for understanding the process of porous flow in the subsurface. A greater understanding of subsurface fluid movement allows for better-informed decisions to be made regarding these areas of research. The large range of applicability that a well-constrained porosity–permeability relationship has highlights the value in working to accurately describe it. Investigation of this relationship with regards to the influence of a material’s grains has traditionally been performed using very laborious and time-consuming techniques which are also destructive in nature. Materials must be crushed and sieved through many incrementally finer sieves to determine a size distribution. Specific size measurements may be performed using callipers, requiring far more time than using an automated digital approach capable of measuring many parameters at the same time. The inclusion of an investigation into a digital, easily repeatable, time effective and accurate technique for constraining the porosity–permeability relationship using grain characteristics in this work is therefore of great value. Overall, the identification of a reliable, accurate and repeatable relationship between porosity and permeability using micro computed tomography (μCT) imaging could have far-reaching benefits to many fields of research and industrial activity.

### Modelling a porosity–permeability relationship

The Kozeny–Carman (K–C) relationship, proposed by Kozeny^1^ and later modified by Carman^2^, is a simple yet broadly effective and widely used^3–7^ technique of relating porosity to permeability. Bear^8^ suggested a modification to the K–C equation which allows grain diameter to be employed as a component which influences the permeability. Additionally, Hommel et al.^7^ and Rasaei and Firoozpour^9^ show that versions of the K–C equation may be used with and without grain sphericity and grain size terms. Whilst a K–C-based approach is successful in many instances, its accuracy may be questioned when applied to materials which possess a significant proportion of grains which deviate substantially from being entirely spherical. The limitation of a K–C approach is that grains are considered spherical and packed in a regular arrangement; allowing pores to be considered as capillary bundles. The inherent relationship between the pore structure and the grains which create the pore space indicates that a detailed investigation of grain characteristics is of utmost importance in understanding the porosity–permeability relationship.

This work aims to investigate whether the inclusion of grain sphericity and 3D Feret diameter (referred to herein as grain size) in a K–C paradigm facilitates a better-quality fit to the relationship between porosity and permeability. A modified K–C approach is compared to a simpler fit using porosity measurements alone, excluding any influence of grain shape or size. To do so, the individual relationships between porosity and permeability and grain sphericity and size are investigated and considered in light of the concept of grain anisotropy, as introduced by Nabawy^10^.

### Digital approaches to making 3D grain measurements

Whilst grain size and shape measurement has traditionally been done manually using callipers and sieve analysis^11–14^, this work uses digital image analysis (DIA) to segment individual grains in 3D using reconstructed X-ray micro computed tomographic (μCT) image stacks of each sample. μCT imaging has been used in a wide variety of fields related to geoscience since its rise in popularity as a non-destructive and high resolution image acquisition technique^15–19^. When paired with DIA, large amounts of quantitative and visually useful data may be obtained. Unlike when using optical imaging, X-ray imaging is dependent primarily on phase density therefore, grain boundaries are difficult to identify, particularly in a tightly packed sandstone.

This work discusses and investigates grain segmentation using two relatively simple marker-based watershed workflows. Watershed algorithms, established by Beucher and Meyer^20^, split a phase up into individual components by treating the image as a topographic surface, identifying topographic lows and assigning a seed point marker to each. Flooding from each marker allows digital watersheds to be identified and used to define the boundaries between individual features^21^. The challenge arises from making correct identifications of marker points so as not to have multiple grains sharing one marker (undersegmentation) or the opposite where multiple markers are assigned to a single grain (oversegmentation). Techniques such as the bring up^22,23^ and bring down^21,24^ methods have been developed to try and tackle this issue but can often be computationally demanding and may still produce inaccuracies.

Segmentation of the solid phase alone allows identification of individual grains which can then be measured digitally in 3D. Segmentation is arguably the most important and usually most difficult process in DIA^25^ given that poor segmentation will directly result in poor and likely misleading results. It is notoriously difficult to segment features within a given phase which are touching, consequently many techniques have been developed to tackle this challenge, often providing unique solutions to a given sample set or type of sample (shelly, angular, rounded, etc.)^22,23,25,26^ as there is no one size fits all solution^25^.

It is often the selection of seed points and the size of markers within watershed segmentation which determines how effective the result is. Other techniques have been developed which attempt to move away from this reliance such as the stochastic watershed using a probability density function^27^. Other approaches include modifying the distance map on which watershed algorithms rely to influence the size of seed points relative to the features which they represent^28^. More recently the use of machine learning approaches during segmentation^29,30^ or in post processing^26^ have also been investigated. These approaches are often complex in nature or their implementation and may involve many steps or machine learning model training. In this work we aim to investigate the use of relatively simple approaches to 3D grain segmentation and measurement using a traditional watershed algorithm, improving the outcome using variations in image pre-processing.

We focus on two similar grain segmentation workflows presented by Fei et al.^31^ and Thomson et al.^32^. The Fei et al.^31^ approach is proven to be successful in segmenting individual 3D grains in μCT images of loosely packed grains of varying angularity which have been extracted as loose sand from geological materials. This means that the resulting μCT images have relatively few contact points between grains. This results in greater accuracy in the segmentation of individual grains. Meanwhile, the Thomson et al.^32^ approach is applied to μCT images of consolidated sandstone core plugs but encounters issues with under and oversegmentation of individual grains. This issue arises from the highly compacted nature of the sandstone core material, relative to loose sand grains, which results in many more closely packed grain boundaries to be distinguished from one another.

These two workflows differ in their image filtering prior to segmentation. Thomson et al.^32^ use a non-local means (NLM) filter to remove any noise and improve the image quality prior to segmentation. NLM filtering is a popular and effective image filter and is often used as the initial step for cleaning up μCT images prior to segmentation^33,34^. Similarly, Fei et al.^31^ use a NLM filter but also apply a median filter afterwards. The median filter is effective in smoothing blemishes within images and emphasising feature boundaries. We believe that the addition of a median filter has the potential to alleviate the severity of over and undersegmentation experienced by Thomson et al.^32^.

The goal of this work therefore is to determine an effective yet simple individual grain watershed segmentation workflow which is capable of operating on compacted grains within core material. This is done by assessing whether the Fei et al.^31^ approach is as effective when applied to compacted rock volumes as it is when applied to loose sand grains. This is determined by comparison with the similar Thomson et al.^32^ approach, used as an acceptable baseline technique for facilitating measurements of individual sandstone grains from core material.

These two segmentation workflows are assessed and the most effective is used to analyse a collection of 22 sandstone samples from three different geological formations (i.e., Wilmslow Sandstone Formation, Sellafield, UK; Brae Formation Sandstone, Miller Field, North Sea, UK; Minard Formation Sandstone, Porcupine Basin, North Atlantic Ocean). Finally, the grain measurements are used alongside digital measurements of porosity and permeability to investigate the quality of a K–C-based fit to the porosity–permeability relationship using grain sphericity and size inputs.

## Methods

A variety of sandstone samples have been selected from several different reservoir units which host significant levels of porosity. Samples from the Wilmslow Sandstone Formation (Sellafield, UK)^17^, Brae Formation Sandstone (North Sea, UK)^18^ and the Minard Formation of the Porcupine Basin (North Atlantic Ocean) were acquired and imaged at the London Natural History Museum Imaging and Analysis Centre. A summary of the materials used in this work can be found in the Supplementary Information (Table S1). Samples which exhibited no connected porosity and therefore no permeability were excluded for the purpose of this study.

The material pertaining to the Porcupine Basin was collected and prepared using the same technique outlined by Thomson et al.^18^ and Payton et al.^17^. From each sample a mini plug measuring 5 mm in diameter and 10 mm in length was cut and imaged using X-ray micro computed tomography (μCT), detailed by Payton et al.^17^. For further information about the voxel size and subsampled volume of each sample the reader is referred to the Supplementary Information (Table S2).

### Image processing

The acquired μCT image stacks of each sample underwent pre-processing using the commercial software package PerGeos (v1.7.0). From each image stack a sub-volume was extracted to remove external voxels and any image slices which contained significant beam hardening artefacts. In order to aid the segmentation process a non-local means filter was employed which enhances the contrast between greyscale phases and removes speckled noise throughout the images^33,34^.

### Porosity and permeability

The method detailed by Payton et al.^17^ was followed to measure porosity and permeability—a brief outline is described here. The well-known automatic binary segmentation algorithm designed by Otsu^35^ was used to separate and label the solid grain phase and pore space. In some cases, it was necessary to constrain the greyscale range over which the algorithm was allowed to operate on where exceedingly bright phases were present which meant darker grains and darker pore space were not automatically separated.

The volume fraction of the segmented pore space can be measured which equates to the total sample porosity. The ‘axis connectivity' tool was applied along each axis in turn to determine the proportion of porosity which is entirely connected between all faces of the sample. This value was taken to represent the connected porosity.

Finally, the ‘absolute permeability simulation' tool was used to run a finite volume numerical simulation, solving the Stokes flow equations:

1 $$\nabla {\varvec{u}}=0$$

2 $$- \nabla P+\mu {\nabla }^{2} {\varvec{u}}=0$$

where ${\varvec{u}}$ is velocity, $P$ is pressure, $\mu$ is fluid viscosity equal to $1\times {10}^{-3}$ Pa s for water. An error tolerance of ${10}^{-6}$ for the convergence of the L_2_ norm of the residuals was implemented as recommended by Thomson et al.^36^ whilst the boundary conditions used are discussed in detail by Thomson et al.^19^. The solution is a velocity field which allows for a permeability value to be determined through application of Darcy's Law. Further details on this technique can be found in Thomson et al.^18^ and Payton et al.^17^.

### Pore geometry

Pore network models (PNMs) were employed to characterise the individual pores which make up the pore structure. PNMs are simplified representations of complex pore geometries using balls to represent pores and sticks to represent throats. PNMs were created of the connected porosity following the methodology detailed by Payton et al.^17^ and references therein. Each PNM may be interrogated to provide information about each pore including radius and coordination number, and each throat including radius and length.

### Individual grain segmentation

**Figure 1:**
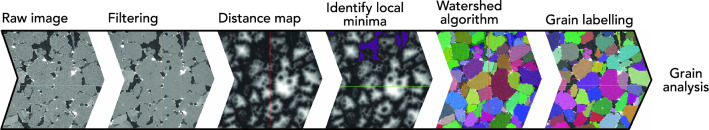
Schematic diagram showing the typical steps in grain identification using a watershed technique on CT images. Figure created using Fiji 2.1.0^42^ and PerGeos 1.7.0: https://www.thermofisher.com/uk/en/home/electron-microscopy/products/software-em-3d-vis/pergeos-software.html.

Segmentation of individual features in μCT images has traditionally been performed using the marker-based watershed approach detailed by Beucher and Meyer^20^. This technique has been widely used in a variety of fields^37–41^ to identify and split individual features in digital images. The general steps in using a watershed algorithm are shown in Fig. 1 (for a more detailed description of how a watershed algorithm operates the reader is referred to Kong and Fonseca^22^ and Sun et al.^21^). Whilst Fig. 1 shows the process in 2D, the outcomes presented using this technique in this work are three dimensional, as shown in Fig. S1. The workflow of watershed segmentation of grains described by Fei et al.^31^ was chosen as it is proven to show effective individual grain segmentation, analysed and evaluated in the Discussion.

The method described by Fei et al.^31^ uses the software package Fiji^42^ to carry out cropping and filtering. A non-local means filter is used in combination with a median filter prior to using the MorphoLibJ plug-in for Fiji^43^ which encompasses generation of a distance map and identification of seed points and markers for watershed flooding as described in Fig. 1.

### Grain measurements

**Figure 2:**
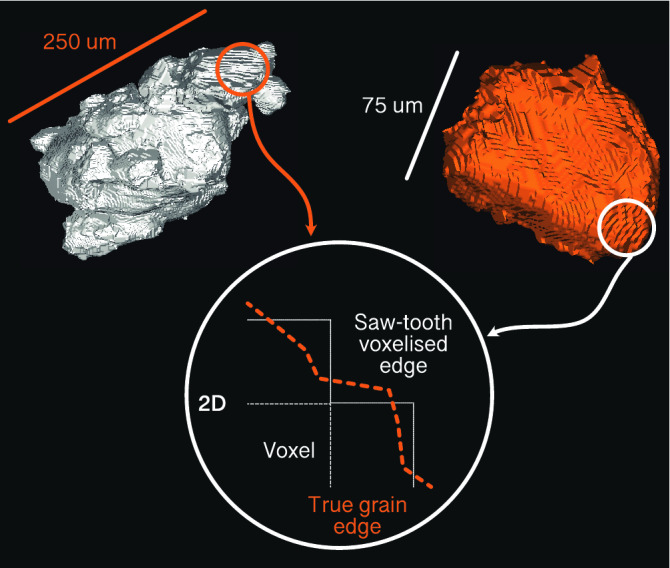
Isolated collection of grains (white) and single grain (orange) shown in 3D from sample SF696. The saw-tooth or staircase pattern is highlighted which arises from the voxelised images. This can lead to overestimation of surface area and impact the subsequent sphericity measurements. Figure created using Fiji 2.1.0^42^.

Once the watershed algorithm has run, the individual grains are labelled and the pore space subtracted before the Feret diameter and sphericity of each grain is measured using the 3D ImageJ Suite plug-in^44^. When extracting 3D grains from μCT images, which are voxelised, the edges exhibit a saw-tooth pattern (Fig. 2). This can lead to overestimation of surface area and consequently underestimation of sphericity, as detailed by Fei et al.^31^. Therefore, it is acknowledged that measurements of sphericity are conservative but as the saw-tooth pattern effect is present for all grains measured, the results are still directly comparable between each other.

Whilst smoothing algorithms can be applied to reduce this effect, determining appropriate parameters for such algorithms becomes heavily subjective and can cause undesirable deformation of the individual grains such as volume loss. Moreover, using the same degree of smoothing on a very small and a very large grain will have different impacts on the resulting shape. Consequently, the use of any smoothing tools prior to measurements being made was omitted.

**Figure 3:**
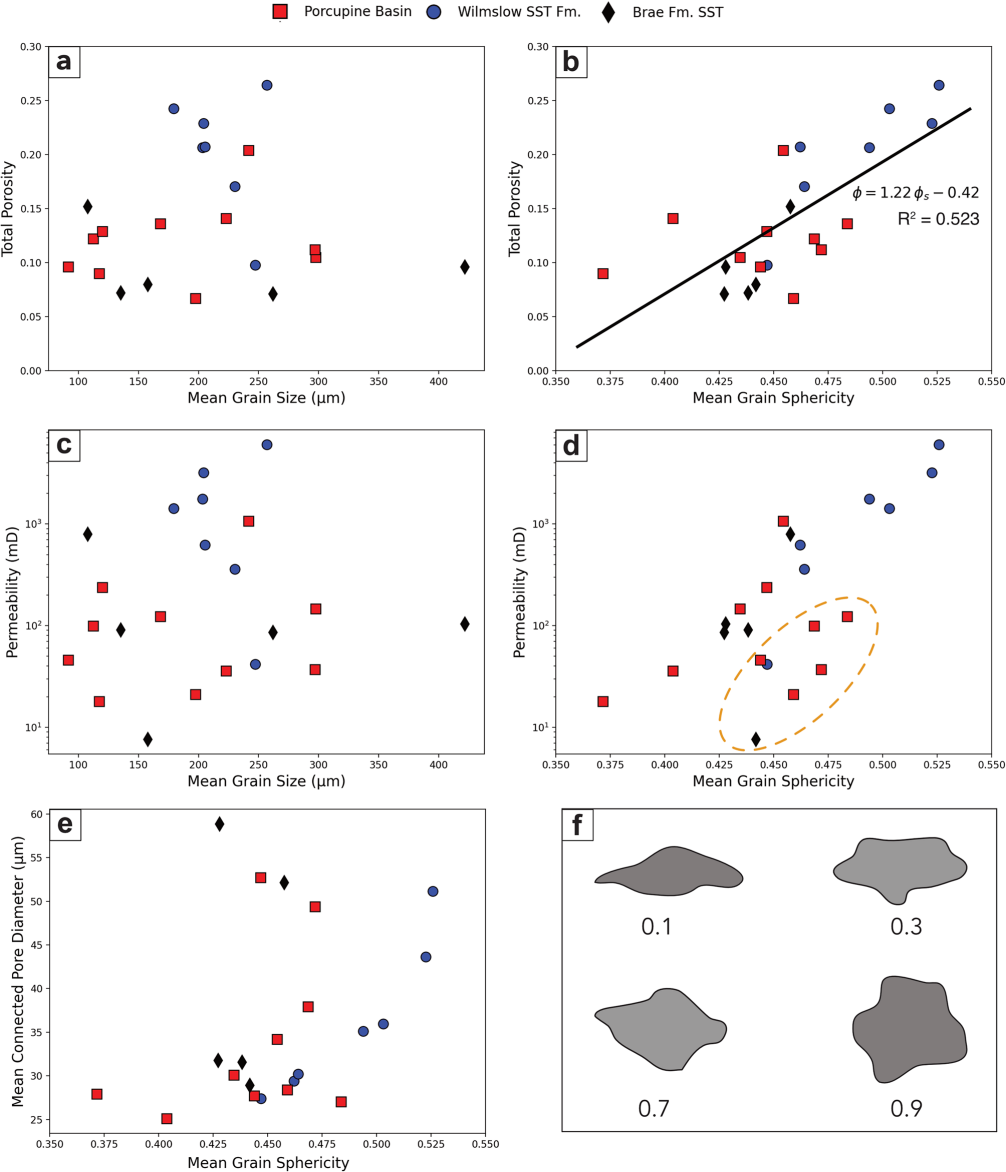
Relationship between mean grain size, mean grain sphericity and total porosity, permeability and mean connected pore diameter. Grain sphericity is measured on a scale between 0 and 1 where 1 is entirely spherical, a schematic diagram is shown to represent the range of sphericities according to Krumbein^46^ plotted in (**f**). A generally positive relationship with porosity and permeability can be observed in the case of mean grain sphericity in (**b**) and (**d**), but no such relationship is present with mean grain size in (**a**) and (**c**). A region of outliers is identified by a dashed line in (**d**) with the same data points also apparent in (**b**) to a lesser extent. A simple linear fit is calculated between grain sphericity (φ_s_) and porosity (φ), defined by the black line and accompanying equation in (**b**). It is apparent that there is a generally positive relationship between sphericity and connected pore diameter aside from a small group of four outliers (**e**).

The automated nature of the MorphoLibJ and 3D Suite plug-ins enables this analysis to be carried out simply as well as rapidly with low computational cost. Sphericity is measured between 0 and 1 where 1 represents a perfect sphere, shown schematically in Fig. 3f. Feret diameter was used as the representative grain size for all statistical analyses in this work. Some of the grain size analyses performed use phi ($\phi$) units, calculated from grain size values in millimetres according to:

3 $$\phi = -{\mathrm{log }}_{2}D$$

where $D$ is the grain diameter. The graphic mean grain size (${M}_{Z}$) was calculated after Folk^45^, according to the following formula:

4 $${M}_{Z}= \frac{(\phi 16+\phi 50+\phi 84)}{3} ,$$

where $\phi 84$ represents the $\phi$ value at the 84th percentile. The ‘inclusive graphic standard deviation' introduced by Folk^45^ was calculated to determine the sorting (${\phi }_{1}$) of the samples using the following formula:

5 $${\phi }_{1}= \frac{\phi 84-\phi 16}{4}+ \frac{\phi 95-\phi 5}{6.6} .$$

Classification of the samples according to sorting was performed following the accompanying scheme defined by Folk^45^ where a smaller ${\phi }_{1}$ value is representative of better sorting.

## Results

The following results were acquired using the methodology described by Fei et al.^31^ which was determined to be the most effective approach for these samples. An evaluation and comparison of the Fei et al.^31^ and Thomson et al.^32^ methods are given in the Discussion, justifying our choice of segmentation approach.

### Application of the proposed methodology

Each study sample was analysed in terms of grain characteristics with the results reported in Table 1. The accompanying porosity and permeability results are reported in Table 2, measured in this article and by Thomson et al.^18^ and Payton et al.^17^. Figure 3 shows the relationships among mean grain size, mean grain sphericity, porosity, and permeability. No clear relationship between grain size and sample porosity or permeability is observed (Fig. 3a,c). Despite this, a much clearer positive correlation between the grain sphericity and porosity and permeability can be seen (Fig. 3b,d). This suggests that the shape of the grains has a direct influence on the pore structure whereas the size of the grains does not. Figure 3d highlights a collection of seven possible outliers showing the same relationship but offset from the dominant trend between mean grain sphericity and permeability. The same collection of data points is highlighted in Fig. 3b, plotting mean grain sphericity against total porosity, where they are not obviously misaligned with the rest of the data points. This indicates that these apparent outliers, in the case of permeability, result from a characteristic of the sample which is independent of porosity but not permeability.

**Table 1:** Grain-based measurements made for each sample.

Sample	Sorting (φ)	Mean grain size (μm)	Mean grain sphericity
PB01	0.63	242	0.45
PB02	0.61	298	0.43
PB03	0.44	112	0.47
PB05	0.45	297	0.47
PB06	0.55	198	0.46
PB07	0.45	92	0.44
PB08	0.42	168	0.48
PB10	0.49	120	0.45
PB11	0.78	223	0.40
PB12	0.56	117	0.37
SF696^a^	0.61	203	0.49
SF697^a^	0.54	205	0.46
SF698^a^	0.64	204	0.52
SF699^a^	0.50	257	0.53
SF700^a^	0.51	230	0.46
SF701^a^	0.51	179	0.50
SF702^a^	0.52	247	0.45
BFS1^b^	0.61	135	0.44
BFS2^b^	0.75	262	0.43
BFS4^b^	0.69	158	0.44
BFS5^b^	0.53	421	0.43
BFS8^b^	0.44	108	0.46

^a^Payton et al.^17^.

^b^Thomson et al.^18^.

**Table 2:** Porosity and permeability measurements made for each sample.

Sample	Total porosity (%)	Connected porosity (%)	Permeability (mD)
PB01	20.4	20.3	1070
PB02	10.5	9.8	147
PB03	12.2	10.2	99
PB05	11.2	4.9	37
PB06	6.7	5.2	21
PB07	9.6	8.9	46
PB08	13.6	13.3	123
PB10	12.9	9.7	237
PB11	14.1	13.6	36
PB12	9	6.9	18
SF696^a^	20.7	20.4	1760
SF697^a^	20.7	20.3	620
SF698^a^	22.9	22.7	3190
SF699^a^	26.4	26.3	6040
SF700^a^	17.0	16.6	360
SF701^a^	24.3	24.1	1420
SF702^a^	9.77	8.89	40
BFS1^b^	7.2	5.8	91
BFS2^b^	7.1	5.7	86
BFS4^b^	9.6	9.1	104
BFS5^b^	7.8	5.1	6.7
BFS8^b^	15.2	14.8	795

^a^Payton et al.^17^.

^b^Thomson et al.^18^.

As the intergranular porosity is fundamentally governed by the grains themselves, the relationship between the pore structure and grain sphericity was investigated. Figure 3e shows a generally positive relationship between grain sphericity and the connected pore diameter, except for four apparent outliers across all three sample suites. Of these four outliers, two belong to the group of seven identified in Fig. 3d and two do not. However, the cause for the occurrence of these four outliers is unclear and it seems that there is no correlation between these four outliers and other measured factors such as sorting and grain size.

### Impact of grain characteristics on the porosity–permeability relationship

The results show that the sphericity of the grains in a sample has an impact on the porosity and permeability. Therefore, it is reasonable to assume that the porosity–permeability relationship could be better constrained through incorporating the grain sphericity into the fit equation. Three variations of the Kozeny–Carman equation were employed in order to fully investigate the influence of grain characteristics on the porosity–permeability relationship. In order to investigate the impact of both grain sphericity and size, a modified Kozeny–Carman equation was employed, discussed by Hommel et al.^7^,

6 $$K=\frac{{\phi }_{s}^{r} {\phi }^{n} {D}_{p}^{2}}{180{\left(1-\phi \right)}^{2} },$$

which incorporates the grain sphericity, ${\phi }_{s}$ and size, ${D}_{p}$ alongside porosity, $\phi$ to calculate a porosity–permeability fit. To investigate the effect of grain size on the relationship the following equation discussed by Rasaei and Firoozpur^9^ was used,

7 $$K= \frac{{\phi }^{n} {D}_{p}^{2}}{180{\left(1-\phi \right)}^{2}}.$$

Finally, to investigate the effect of grain sphericity on the porosity–permeability relationship the following equation was employed where ${K}_{0}$ is a constant,

8 $$K={K}_{0} \frac{{\phi }_{s}^{r} {\phi }^{n}}{180{\left(1-\phi \right)}^{2}}.$$

**Figure 4:**
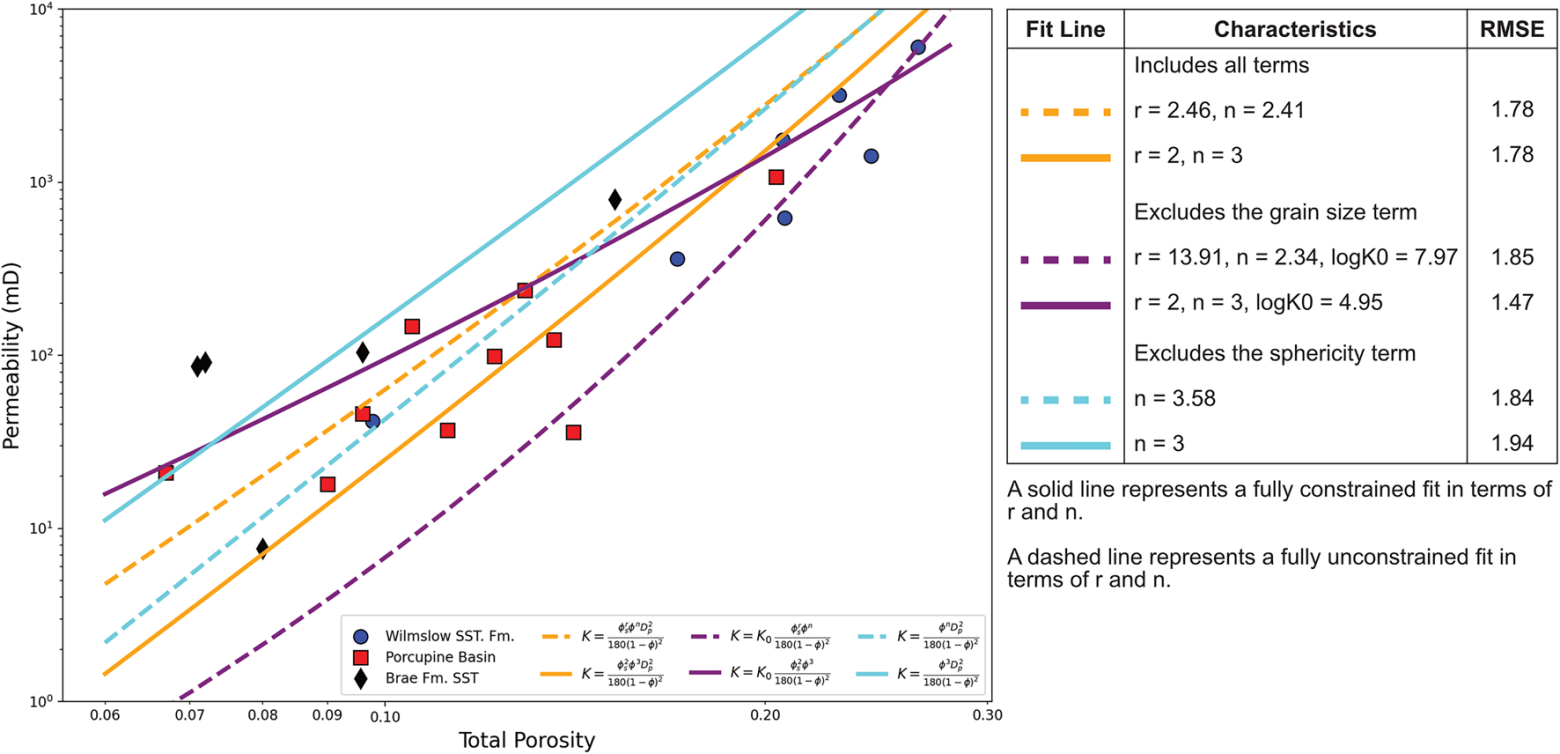
Range of calculated fit configurations to the porosity–permeability relationship which incorporate grain characteristics using Kozeny–Carman based relationships. The table to the right describes the difference between each fit line and reports the values of the exponents whilst the respective equations are displayed in the plot legend.

In the case of each fit, the exponents $n$ and $r$, applicable to porosity and grain sphericity respectively (Fig. 4), were either constrained according to the values given in the respective literature or allowed to vary. Comparison between the fit lines produced using these three equations allows for the influence of each grain parameter to be determined. This approach was used to determine the best fit with the lowest root mean square error (RMSE).

The best of the four fits based on the RMSE (Fig. 4) is the case where grain size is excluded and the exponents are constrained as given in the literature. The poorest quality fit is produced when the grain sphericity term is omitted and the porosity exponent is constrained. These observations align with the observations made in Fig. 3. Omission of grain size, which is shown to have no relationship with permeability or porosity, produces the best fit. Meanwhile exclusion of the sphericity term, which has a positive relationship with porosity and permeability, produces the poorest fit.

The other fit lines displayed in Fig. 4 exhibit similar RMSE values to the poorest fit. In the case of the orange and blue fit lines this attests to the inclusion of a grain size term, causing detrimental effects on the fit qualities. Meanwhile, the also poor RMSE of the unconstrained fit, which excludes grain size, attests to the value in constraining the exponents according to the literature.

**Figure 5:**
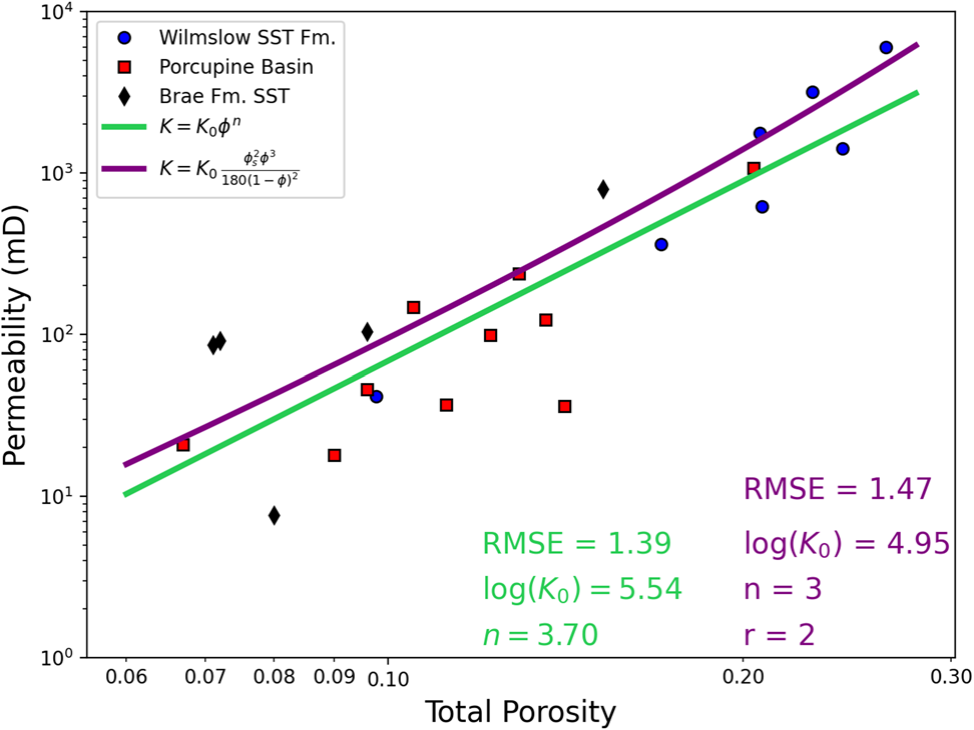
Calculated fits to the porosity–permeability relationship displayed on log–log axes. A simple fit is shown (green) alongside the most effective fit incorporating grain sphericity (purple) shown in Fig. 4. The root mean square error (RMSE) values are reported for each fit, showing that the better fit is the simpler one in green, excluding any grain characteristics.

It is apparent that even the best fit achieved, shown by the solid purple line in Fig. 4 does not fit all data points effectively, especially below a total porosity of ca. 15%. Consequently, an additional, simpler fit is shown which does not consider any grain characteristics in Fig. 5 (green line) alongside the best fit identified in Fig. 4. The results show that the simpler fit which considers porosity and permeability alone is slightly more effective, exhibiting a lower RMSE of 1.39 as opposed to 1.47 in the case of the fit incorporating grain sphericity.

## Discussion

**Figure 6:**
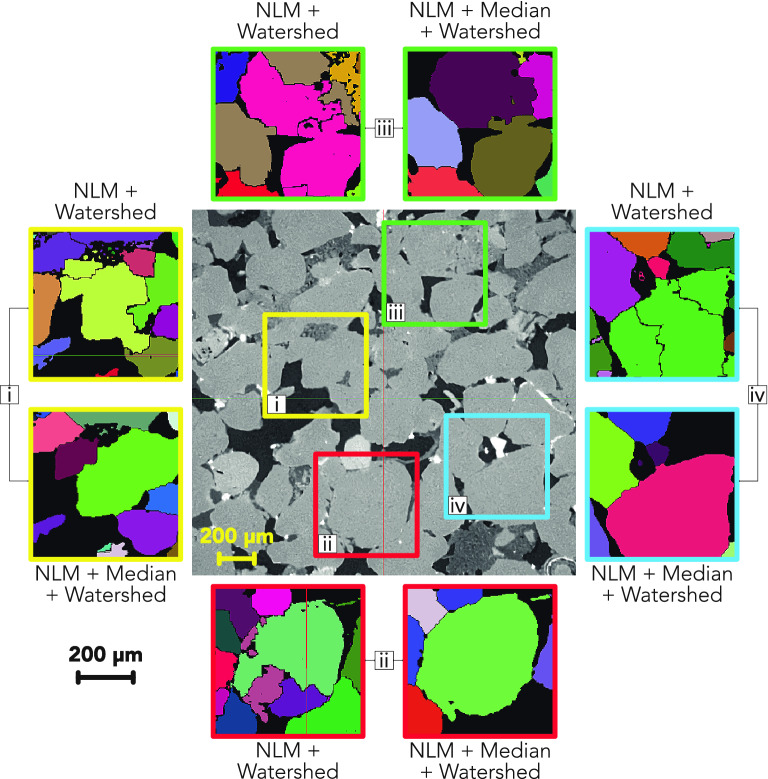
Comparison of two different filtering techniques' effects on the watershed algorithm in a single slice of sample PB10. Four different locations have been highlighted for comparison on an image which has undergone non-local means (NLM) filtering only. Annotated squares show the result of watershed grain segmentation following only NLM^32^ and NLM with a median filter^31^. Each grain can be identified by a different colour however, due to the number of grains, colours have been reused and instead the black grain boundaries split different grains of the same colour. In each annotation an example of over-segmentation is observed in the case of using NLM filtering only when compared to what might be expected from the CT image. The outer scale bar applies to all annotations. Figure created using Fiji 2.1.0^42^ and PerGeos 1.7.0: https://www.thermofisher.com/uk/en/home/electron-microscopy/products/software-em-3d-vis/pergeos-software.html.

Whilst the two compared approaches chosen to segment individual grains^31^ are relatively straightforward, it is acknowledged that there are a number of other algorithms which aim to improve the accuracy of the traditional watershed algorithm. In particular, oversegmentation is an issue, particularly when segmenting features of a wide range of sizes and shapes where multiple markers are placed within one feature^21–23^. Modified watershed approaches have been developed using the bring down method^24^ and the bring up method^22,23^ to accurately label features and their boundaries. Other techniques based on machine learning and modification of the distance map have also been developed but are consequently more complex and often require model training^26,28–30^. Due to the high accuracy of results reported by Fei et al.^31^ alongside the quality of segmentation observed in Fig. 6, the ease of implementation and minimal computational cost the traditional watershed technique was chosen with a non-local means and median filter in line with the methodology described by the authors.

The technique used here is very similar to that applied by Thomson et al.^32^. Thomson et al.^32^ implement a traditional watershed algorithm but only use a non-local means filter without a median filter. The non-local means filter performs the bulk of the denoising in the images very effectively, but this type of filter is not optimal for retaining or improving feature boundaries. In contrast, the median filter is very effective for this purpose, enhancing the clarity of feature boundaries whilst smoothing any remaining noise in the images. The similarities and differences in the results of watershed segmentation using the two approaches are shown in Fig. 6. We refer the reader to the Supplementary Information (Fig. S1) to see a 3D representation of the grain segmentation carried out in this study.

The results show that the approach used by Thomson et al.^32^ results in some oversegmentation of grains when comparing the watershed result to the greyscale CT image. In contrast the approach used in this study does not show severe oversegmentation of the same grains, owing to the boundary enhancement provided by the median filter. Furthermore, by using the 3D Suite plug-in for Fiji, grains which are touching the boundaries of the study volume can be excluded from measurement to ensure only grains which are complete and truly representative are included. This was not included in the method used by Thomson et al.^32^ and therefore partial grains may have significantly influenced the mean grain measurements made.

Finally, Thomson et al.^32^ acknowledge in their work that the separated grains in their work displayed an unexpected group of grains with Feret diameters of < 63 μm, smaller than the classification of sand grains following the scheme proposed by Wentworth^47^. Employing the additional median filter largely removed the occurrence of these small, unexpected grains. Therefore, this suggests that the combination of a median filter with a non-local means filter is effective in reducing over segmentation and identification of small, unexpected features.

Application of this segmentation technique revealed the lack of relationship between mean grain size and both porosity and permeability (Fig. 3a,c). This strongly suggests that grain size within this suite of samples is not influential on the porosity–permeability relationship of the respective pore structures. Nabawy^10^ presents a similar conclusion when examining the influence of grain size on porosity and permeability in a series of idealised grain packs as well as in high porosity sandstone samples. All but two of the samples are classified as very well-, well- or moderately-sorted^45^. Therefore, it is suggested that future work should focus on the relationship between grain size and porosity and permeability in a variety of sandstones of different grain maturity, shape, sorting and facies to identify any factors which may influence whether grain size presents a relationship with porosity or permeability.

In contrast, evidence that mean grain sphericity has a direct positive impact on both porosity and permeability is shown in Fig. 3b,d. Nabawy^10^ identifies a similar relationship with the elongation (grain length/grain diameter) of grains within their sample suite where less elongate grains contribute to greater porosity and permeabilities. Nabawy^10^ uses elongation as a measure of grain anisotropy where a more elongate grain indicates a greater degree of anisotropy. The same approach can be applied to grain sphericity, where a less spherical grain indicates a greater degree of anisotropy. Following this paradigm, the results presented here agree with those of Nabawy^10^, a greater degree of anisotropy of the grains results in a reduction in both porosity and permeability.

Despite the clarity of the relationships between sphericity and porosity and permeability in Fig. 3, it is important to consider the range of sphericities exhibited by the study samples. All samples exhibit sphericities between 0.35 and 0.55. It is possible that the relationship demonstrated here shows variation outside of this sphericity window. Therefore, we suggest that future work should focus on examining this relationship over a wider range of sphericities using additional study samples.

A simple linear fit was calculated for the relationship between mean grain sphericity and total porosity which is given by $\phi =1.22 {\phi }_{s}-0.42$. Nabawy^10^ proposes a relationship between elongation, $E$ and porosity using their sample suite where $\phi =45.73 {E}^{-1}+9.19$. This provides two parameters by which a porosity estimation may be made based upon two different measures of grain anisotropy. Whilst Nabawy^10^ achieves an elongation fit exhibiting a correlation coefficient of 0.92, the sphericity fit presented here has a correlation coefficient of 0.72. This work considers three separate sample suites from different sedimentary facies, whilst Nabawy^10^ focusses on a single sample suite, which makes the relationship between anisotropy and porosity less clear. Consequently, it is suggested that different depositional environments may have a more significant effect upon the characteristics which influence the relationship between grain anisotropy and porosity, as opposed to there being one consistent relationship being applicable across a wide variety of sandstones. Further research is required to quantify the scale of this influence.

The control which the anisotropy of grains has on the geometry of the pores themselves is also investigated, finding that there is generally a positive relationship between grain sphericity and pore diameter (Fig. 3e), aside from the four described outliers. The results agree with the relationship identified between porosity and grain anisotropy, measured through elongation^10^. This indicates that these two measures of grain anisotropy exhibit similar controls on porosity which reflects directly in the geometry of the pore structures.

A suggested limitation of the relationship reported by Nabawy^10^ is that it may depend on grain elongation occurring systematically along one axis which is common throughout the sampled well-sorted material. Such imbrication of grains according to their elongation axes is likely to result due to the flow of depositional currents and load pressure. Where such an alignment is not clearly present, for example under depositional conditions where turbulent flow dominates, these results imply that the detrimental impact on permeability would be far more pronounced than any influence on the relationship with porosity. This conclusion requires further testing using samples from varied depositional environments prone to varying degrees of imbrication to establish if this is a key influencing factor.

A group of seven possible outliers is observed when examining the relationship between grain sphericity and permeability (Fig. 3d) which fall below the dominant trend. The fact that this group of outliers are not apparent when comparing sphericity with porosity (Fig. 3b) strongly suggests that their rogue placement is due to a factor which inhibits fluid flow but does not change the absolute porosity measurement. This may point towards a lack of preferential orientation with regards to grain anisotropy within these particular samples, relative to the majority of the study samples.

Further investigation of the seven outliers found that there was no apparent common characteristic amongst them which could differentiate them from the remaining samples. It was investigated whether there was a relationship between these outliers and their sample depth. The thickness of the overburden may have influenced grain alignment and consequently permeability in each study sample as a result of compaction. However, we found there to be no relationship between sample depth and porosity or permeability across all study samples. It has been noted that grain packing textures have significant control on porosity reduction during compaction^48^ which would then have an influence on permeability. It is possible that the outlier samples possess unique packing textures compared to the remaining samples which could explain their deviation from the majority of the study samples. We recommend further investigation into the influence of grain packing textures on permeability in these materials and others. Sorting, porosity and permeability were also investigated with regards to the outliers but no relationship was found which might explain their occurrence. None of these characteristics helped to explain the presence of the seven outliers. Furthermore, a qualitative assessment of the μCT images found nothing of significance which might allow for the differentiation of this sample group such as presence of cement or other precipitates which were not present in the main group of samples.

It might be expected that a lack of grain orientation would manifest throughout a given geological unit, leading to surprise that the outlier group contains at least one sample from each of the three studied formations. It is suggested that the resulting texture may be controlled by a different depositional process. Alternatively, the scale of the sample upon which measurements were made could be considered not suitably representative for the scale of the processes which cause variation in grain imbrication and alignment with regards to anisotropy. Therefore, it is recommended that future work should focus on identifying a suitable representative elementary volume over which measures of grain anisotropy, such as elongation and sphericity, can be representatively measured. We also suggest that future investigations could use samples taken in different orientations of the same facies to further investigate effective representation of grain orientation. Equally, identification and implementation of a technique to measure and quantify alignment or imbrication of grains in 3D at the pore scale would be beneficial in providing greater context for relationships between porosity and permeability with measures of grain anisotropy.

Despite the positive relationship identified between mean grain sphericity and porosity and permeability (Fig. 3b,d), it has been found that the influence of grain characteristics is not beneficial to constraining the porosity–permeability relationship in these sample suites (Fig. 5). The lower RMSE of the simple fit, which omits any parameters based upon grain characteristics, suggests that grain characteristics are not more effective in constraining the porosity–permeability relationship than porosity alone. It is very apparent from Fig. 4 that including grain size is heavily detrimental to the quality of fit, in agreement with Fig. 3. However, even when grain size is omitted and sphericity is included the fit is still poorer than the simple fit, despite the positive relationship observed between sphericity and porosity and permeability (Fig. 3).

Whilst consideration of grain characteristics is shown to be detrimental in the range of study samples analysed here, further investigation of a wider range of materials would allow it to be determined whether this is representative of all sedimentary materials. The poorer quality of fit determined using grain characteristics may be a result of using a Kozeny–Carman fit equation which makes the assumption that grains are spherical producing a simple pore structure^49^. Bear^8^ describes how this assumption arises from the transformation of the specific surface area term^2^ to a characteristic grain size term.

Inclusion of grain size in the paradigm of a Kozeny-Carman relationship defines the diameter of the grain which is assumed to be spherical. However, definition of the grain size is given as the greatest distance from one side of the grain to another or the calliper distance, which is applicable to non-spherical grains. Therefore, as the sphericity of a given grain reduces, it moves further from the Kozeny–Carman assumption which results in a poorer fit to samples with a lower mean grain sphericity. It is observed that a lower sphericity results in a lower porosity and permeability (Fig. 3) therefore, it is expected that the Kozeny-Carman fit would be poorer at lower porosities and permeabilities.

It is shown to be the case that lower sphericity or greater grain anisotropy results in a poorer agreement with a Kozeny–Carman based fit incorporating grain parameters (Figs. 4 and 5). It can be observed that below ca. 15% total porosity a greater proportion of data points in Fig. 6 lie below the purple fit line whereas the remaining data points lie closer to the fit line above ca. 15% porosity. Torskaya et al.^50^ investigate the effect of grain shape on permeability and find that when using realistic grain shapes from μCT images that the K–C equation underestimates permeability by between 30 and 70%. When using simplified and spherical grain shapes Torskaya et al.^50^ find that the K–C equation fit was far more successful, supporting the conclusion that the K–C spherical grain assumption is causing the poorer quality fit. The K–C approach used here, therefore, is not suitable for use with materials where grains are significantly non-spherical.

As a result of this identified limitation, it is suggested that future work should look to develop an alternative model which accounts for variation in grain sphericity within and between different sandstone samples. In this study it has been clearly shown that grain sphericity exhibits a strong relationship with both porosity and permeability over the range of sphericities measured in these samples (Fig. 3), highlighting the possible value in incorporating this grain characteristic in a porosity–permeability model. A model which is still able to incorporate each influencing factor as individual terms (as in Eq. 6) would be favourable to provide flexibility and the ability for experimentation. Such a model could be tested against the simple and K–C models presented in Fig. 5 based upon RMSE.

Whilst many modified versions of the Kozeny–Carman equation have been proposed and used^7,51,52^, the fundamental assumption of spherical grains and pores arranged as bundles of capillaries remains. Alternatives to a K–C approach at the same scale have been used to describe permeability such as the Fair-Hatch, Brinkman and Panda and Lake models, described and summarised by Le Gallo et al.^51^ and MacQuarrie and Mayer^52^. Whilst some of these approaches use grain size terms, they do not include terms which allow for direct inclusion of grain shape or anisotropy.

A further consideration which would be highly beneficial to any future model would be to account for the percolation threshold, a key phenomenon which makes effectively characterising the porosity–permeability relationship difficult over a range of porosities. Thomson et al.^18^ and Payton et al.^17^ show the percolation threshold for full connectivity to be at ca. 8–15% total porosity, whilst Mavko and Nur^3^ and Rahrah et al.^49^ show the value of incorporating the percolation threshold into a K–C style fit. Consideration of the percolation threshold alongside variable grain sphericity would surely be an effective approach to best describe the porosity–permeability relationship.

## Conclusions

In this work a comparison has been made of two similar grain segmentation techniques, using marker-based watershed algorithms, for reliable and accurate grain boundary identification across the sample suites. It has been found that using a median filter in addition to a non-local means (NLM) filter prior to segmentation results in superior grain separation as opposed to using a NLM filter alone. This was concluded to be due to the ability of the median filter to preserve and enhance the grain edges during denoising, reducing oversegmentation. The low computational cost, simplicity and high speed at which this technique can be applied, compared to more complex machine learning-based approaches, makes this a suitable option for segmentation of sandstone materials such as those investigated here.

Digital image analysis techniques have been used on μCT images of three different suites of sandstone samples to investigate the impact of grain characteristics on the porosity–permeability relationship. In this collection of samples, the porosity–permeability relationship is not better constrained when including grain shape or size parameters in a Kozeny-Carman type fit equation when compared to a simple fit using porosity measurements alone. This is the case despite identification of a strong positive relationship between grain sphericity and both porosity and permeability over the studied range. No such relationship with grain size was identified. Therefore, it was proposed that the porosity–permeability relationship is best described by $K={10}^{5.54} {\phi }^{3.7}$.

It was determined that the need to assume that grains are entirely spherical when working in a Kozeny–Carman paradigm is severely limiting to identifying an effective porosity–permeability relationship. Future work should focus on incorporating a grain sphericity term in a model which effectively handles non-spherical and non-uniform grains. Of added benefit would be consideration of the percolation threshold in producing a model capable of constraining the porosity–permeability relationship over a range of porosities in sandstones.

Finally, consideration of grain sphericity as a measure of 3D grain shape anisotropy revealed a relationship of decreasing anisotropy resulting in greater porosity and permeability, in agreement with 2D measures of grain anisotropy. Total porosity was found to vary with grain sphericity according to $\phi =1.22{\phi }_{s}-0.42$, offering an additional indirect method of predicting porosity. A group of outliers are identified, vertically displaced below the main trend of the sphericity-permeability data. It is suggested that this may be due to a relative lack of grain orientation with regards to sphericity in these samples, inhibiting the permeability only as the same occurrence is not observed so strongly in the case of porosity.

## Supplementary Information

Supplementary Information.

## Data Availability

The μCT images used in this article are available from a variety of sources. Images of the Wilmslow Sandstone Fm. for samples with a SF prefix are available from Payton et al.^17^, stored in the BGS National Geoscience Data Centre (NGDC). Images of the Brae Fm. Sandstone for samples with BFS prefix are not publicly available and must be requested from Thomson et al.^18^. Images of the Minard Formation Sandstone from the Porcupine Basin for samples with a PB prefix are available from the Royal Holloway, University of London Figshare Repository, https://doi.org/10.17637/rh.16955068.

## Code availability

The code used to perform analysis and plot the output of the DIA measurements is available alongside the DIA measurement outputs in the Royal Holloway, University of London Figshare repository, https://doi.org/10.17637/rh.16940569.
