# Supplementary material for: The influence of grain shape and size on the relationship between porosity and permeability in sandstone: a digital approach
Source: Sci Rep. 2022 May 9;12:7531. doi: 10.1038/s41598-022-11365-8 (PMC9085878; doi:10.1038/s41598-022-11365-8)
Supplement: Supplementary file 1 — Supplementary Information. [file 41598_2022_11365_MOESM1_ESM.docx]

**Supplementary Information**

for the article

**The Influence of Grain Shape and Size on the Relationship Between Porosity and Permeability in Sandstone: A Digital Approach**

published in

**Scientific Reports**

Ryan L. Payton^1,*^, Domenico Chiarella^1^ and Andrew Kingdon^2^

^1^ Royal Holloway, University of London, Department of Earth Sciences, Egham, Surrey, United Kingdom

^2^ British Geological Survey, Keyworth, Nottingham, United Kingdom

* Corresponding author: [ryan.payton.2015@live.rhul.ac.uk](mailto:ryan.payton.2015@live.rhul.ac.uk)

| **Sampling Location** | **Well ID** | **Sample ID** | **Depth (m)** | **Stratigraphic Interval** | |
| --- | --- | --- | --- | --- | --- |
| Porcupine Basin, N. Atlantic | 26/28-1 | PB01  PB02  PB03  PB05 | 2271  2256.4  2420  2420.48 | Minard Formation | Renard Member |
|  |  |  |  |  | Dooneragh Member |
|  | 26/28-2 | PB06  PB07  PB08  PB10  PB11  PB12 | 2117  2118  2116.8  2118.6  2119.15  2119.85 |  |  |
| Sellafield, UK | SFBH13B | SF696  SF697  SF698  SF699  SF700  SF701  SF702 | 63.8  76.1  96.98  126.27  144.03  172.16  181.39 | Wilmslow Sandstone Formation | |
| North Sea, UK | 16/7b-20 | BFS1  BFS2  BFS4 | 4040.1  4041.35  4045.13 | Brae Formation Sandstone | |
|  | 16/7b-23 | BFS5  BFS8 | 4061  4063.75 |  |  |

**Table S1.** Summary of where the study samples were collected from. Samples from Sellafield, UK were collected by Payton et al.^[17]^ and North Sea samples by Thomson et al.^[18]^.

**Table S2.** Summary of the μCT image characteristics of each study volume. Samples from Sellafield, UK were imaged by Payton et al.^[17]^ and North Sea samples by Thomson et al.^[18]^.

| **Sample ID** | **Voxel Size (μm^3^)** | **Study Volume Size (μm)** |
| --- | --- | --- |
| PB01  PB02  PB03  PB05  PB06  PB07  PB08  PB10  PB11  PB12 | 2.519  2.519  2.519  2.519  2.519  2.519  2.519  2.519  2.519  2.519 | 1687.73 × 1687.73 × 2350.23  1697.81 × 1697.81 × 2350.23  1684.54 × 1684.54 × 2354.33  1709.73 × 1709.73 × 2354.33  1709.72 × 1709.72 × 2354.33  1709.73 × 1709.72 × 2354.33  1709.72 × 1709.72 × 2354.33  1709.72 × 1709.72 × 2354.33  1709.72 × 1709.72 × 2354.33  1709.72 × 1709.72 × 2354.33 |
| SF696  SF697  SF698  SF699  SF700  SF701  SF702 | 2.668  2.6861  2.6861  2.6861  2.6862  2.6862  2.8409 | 1590.12 × 2073.59 × 2575.88  1786.26 × 1909.82 × 2575.97  1737.91 × 1960.85 × 2575.97  1866.84 × 1834.61 × 2575.97  1829.3 × 1861.54 × 2576.07  1842.73 × 1850.79 × 2576.07  1971.58 × 1926.13 × 2724.42 |
| BFS1  BFS2  BFS4  BFS5  BFS8 | 1.9727  1.9723  1.9725  3.9448  3.9448 | 1301.98 × 1301.98 × 1775.43  1814.52 × 1814.52 × 1873.69  1420.2 × 1420.2 × 1873.88  2603.57 × 2603.57 × 3550.32  2603.57 × 2603.57 × 3550.32 |


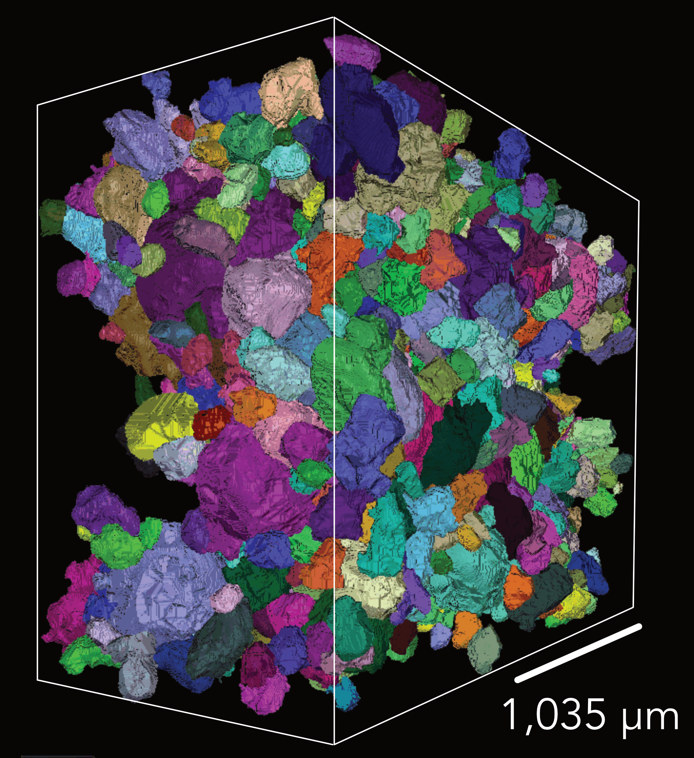


**Figure S1.** Volume rendering of individual segmented grains in sample SF696 following the NLM, median and watershed approach. Each colour represents a different grain, although due to the number of grains colours are reused throughout the figure. Due to the need to exclude incomplete grains there are apparent gaps around the boundaries of the sample volume. Figure created using Fiji 2.1.0^[42]^.

**REFERENCE LIST – In accordance with main body of the article**

17. Payton, R. L. *et al.* Pore-scale assessment of subsurface carbon storage potential: implications for the UK Geoenergy Observatories project. *Pet. Geosci.* **27**, petgeo2020-092 (2021).

18. Thomson, P.-R. *et al.* Pore network analysis of Brae Formation sandstone, North Sea. *Mar. Pet. Geol.* **122**, 104614 (2020).

42. Schindelin, J. *et al.* Fiji: an open-source platform for biological-image analysis. *Nat. Methods* **9**, 676–682 (2012).
